# Comparison of the Mitotic Count With Various Proliferative Markers for the Effective Differentiation of Benign, Borderline, and Malignant Phyllodes Tumors

**DOI:** 10.7759/cureus.71494

**Published:** 2024-10-14

**Authors:** Keigo Amaya, Akira Okimura, Hiroshi Hirano, Midori Wakiya, Hideaki Hirai, Toshitaka Nagao, Takashi Ishikawa, Kimito Yamada, Munehide Nakatsugawa

**Affiliations:** 1 Department of Breast Surgery, Tokyo Medical University Hachioji Medical Center, Hachioji, JPN; 2 Department of Diagnostic Pathology, Tokyo Medical University Hachioji Medical Center, Hachioji, JPN; 3 Department of Anatomic Pathology, Tokyo Medical University, Shinjuku, JPN; 4 Department of Breast Oncology and Surgery, Tokyo Medical University, Shinjuku, JPN

**Keywords:** breast tumor, differentiation, mitotic count, phyllodes tumor, proliferative marker

## Abstract

Introduction

Phyllodes tumors of the breast are categorized as benign, borderline, or malignant based on the WHO classification, which provides comprehensive criteria for determination, such as stromal cell density, stromal cell atypia, mitotic count, borderline status, and presence of ectopic stromal components. The present study was conducted to determine whether there is a proliferative marker superior to mitotic count.

Methods

The cohort comprised 47 benign, 13 borderline, and 16 malignant phyllodes tumors classified according to the WHO criteria. Various cell cycle-related proteins, including Ki-67, Cyclin A2, Cyclin B1, Cyclin E, phospho-histone H3, and Survivin, were used as proliferation markers. Cutoff values, sensitivity and specificity, and positive predictive value (PPV) were determined using the receiver operator characteristic (ROC) analysis.

Results

When comparing sensitivity and specificity using cutoff values to differentiate malignant phyllodes tumors from benign and borderline phyllodes tumors separately, only mitotic count alone showed a value exceeding 0.9. Additionally, the mitotic count showed a PPV greater than 0.8 in all three types of differentiation. However, when differentiating between benign and borderline phyllodes tumors, none of the proliferative indices, including mitotic count, exhibited a value greater than 0.9 for both sensitivity and specificity, and the PPV for borderline tumors did not exceed 0.4.

Conclusion

These findings suggest that mitotic count is the most reliable index for assessing proliferation, for differentiation of malignant from benign or borderline phyllodes tumors. Furthermore, it was revealed that criteria other than the proliferation index play a crucial role in distinguishing between benign and borderline phyllodes tumors.

## Introduction

Phyllodes tumors of the breast comprise spindle-shaped stromal tumors and non-tumor epithelial/myoepithelial cells, with the protruding portion of the stromal mass covered by epithelial/myoepithelial cells, and producing a leaf-like structure with extension [1]. The recent WHO classification divides phyllodes tumors of the breast into three groups: benign, borderline, and malignant [1]. Recurrence is seen in approximately 20% of patients with malignant phyllodes tumors, with a median time of 22.9 months (2.4-72.7 months), and those tumors show rapid growth, with potential for systemic metastasis [2]. Criteria used for the WHO classification are based on stromal cellularity, stromal atypia, stromal mitotic activity, state of the tumor border, stromal overgrowth, and malignant heterogeneous elements [1,2], with cell proliferation activity being a significant factor for classification.

Ki-67, Cyclin A2, Cyclin B1, Cyclin E, phospho-histone H3 (PHH3), and Survivin are known to be mitotic cycle-related proteins. Of those, Ki-67 is expressed outside of the G0 and M1 phases of the cell cycle and is considered to be a useful marker for determining tumor grade [3]. Cyclin A2 is expressed from the S to M phases, Cyclin E from the G1 to S phases, Cyclin B1 in the M phase, and PHH3 in the late G2 to M phases [4-6], while Survivin is expressed from the G2 to M phases [7]. Presently, the expression rate of Ki-67 is universally used to determine the grade of brain and soft tissue tumors [8,9]. While studies have shown the utility of Ki-67 for determining phyllodes tumor grade [10,11], conflicting reports stating that it is not useful have also been presented, and note that consensus has not been reached [12]. Another study presented findings indicating a significantly higher expression of Survivin in malignant phyllodes tumors, though only a few cases were examined [13]. On the other hand, PHH3 expression has been reported as an excellent marker for assessing proliferative activity in phyllodes tumors, showing a strong correlation to mitotic counts [6]. The present study examined mitotic count, expression counts of Cyclin A2, Cyclin B1, Cyclin E, and PHH3, and labeling indices of Ki-67 and Survivin, to determine the best index for proliferation related to the differentiation of malignant phyllodes tumors in the histologically graded phyllodes tumors according to the WHO classification.

## Materials and methods

Ethical statements

The study protocol adhered to the ethical guidelines outlined in the 1975 Declaration of Helsinki and the Ethical Guidelines for Epidemiological Research established by the Japanese government. Prior to commencement, this study received approval from the Institutional Review Board of Tokyo Medical University (No. T2023-0169). The subjected patients and the study contents were disclosed on the website of Tokyo Medical University, and the opportunity was given to the department conducting the study to offer that patients who did not agree with the study be excluded from it. If the department did not receive a request from the patient to be excluded, it was assumed that consent had been obtained. Consent was obtained from patients through the so-called comprehensive consent system.

Enrolled patient

A total of 76 patients with phyllodes tumors treated at Hachioji Medical Center and Tokyo Medical University Hospital between 2006 and 2020 were enrolled. All enrolled patients provided consent for the use of tumor tissues. The tumors included 16 malignant, 13 borderline, and 47 benign phyllodes tumors, with the classification determined by comprehensive judgment based on the WHO criteria (Table 1) [1]. To date, no recurrence has occurred in patients with benign and borderline phyllodes tumors, while recurrence occurred in 4 out of 16 patients with malignant phyllodes tumors, who experienced recurrence within two years after the surgery.

**Table 1:** WHO histological criteria for benign, borderline, and malignant phyllodes tumor.

Histological feature	Benign	Borderline	Malignant
Stromal cellularity	Variable, scant to uncommonly cellular, usually uniform	Cellular, usually mild, may be non-uniform or diffuse	Cellular, usually marked and diffuse
Stromal atypia	None	Mild to moderate	Marked
Mitotic activity	Usually low	Usually frequent	Usually abundant
Stromal overgrowth	Absent	Absent (or very focal)	Often present

Immunohistochemistry of proliferative markers

Following surgical resection, the tumor was immediately cut at the maximum diameter and fixed in 10% phosphate-buffered formalin (pH 7.4). Then, several blocks were made from the maximal cut surface. Each block was cut into several 4-μm-thick sections for hematoxylin and eosin staining, as well as immunohistochemistry for the detection of proliferation markers using an automated system (Leica BOND-III; Leica Biosystems, Nussloch, Germany) with the antibodies listed in Table 2. Antigen retrieval was performed by this automated system according to the ER2 protocol.

**Table 2:** Antibodies used for immunohistochemistry of various proliferative markers.

Antigen	Clone	Source	Dilution
Ki-67	MIB-1	Dako, Glostrup, Denmark	1:200
Cyclin A2	1C6C11	Proteintech, Rosemont, IL, USA	1:500
Phospho-histone H3 (PHH3) (Ser10)	Rabbit polyclonal	Cell Signaling, Danvers, MA, USA	1:200
Cyclin E	Rabbit polyclonal	Biorbyt, Cambridge CB4, UK	1:200
Cyclin B1 (CCNB1)	Rabbit polyclonal	Biorbyt, Cambridge CB4, UK	1:100
Survivin	Rabbit polyclonal	Novus Biologicals, Centennial, CO, USA	1:800

Quantification of mitosis and expression of each proliferative marker

**Figure 1:**
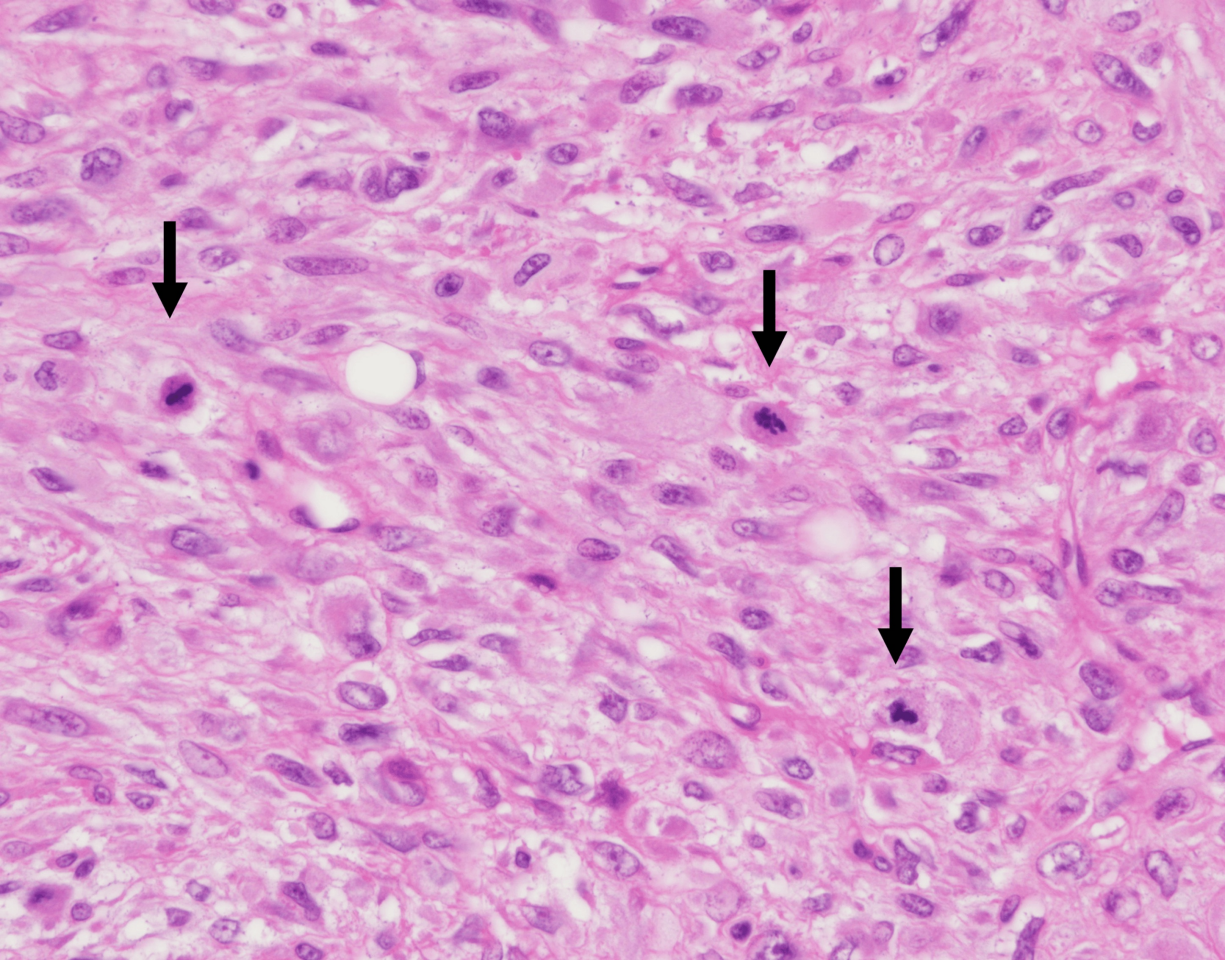
Mitotic figures (arrows) in malignant phyllodes tumor (x40).

Initially, optical microscopy was used to select several sections containing hot spots indicating mitosis or each proliferative marker. The mitotic count was assessed in 10 high-power fields (HPF) of hot spots (total area: 2.38 mm²) using an optical microscope (Nikon, ECLIPSE 80i, x22 eyepiece, 40x objective; Nikon Corporation, Tokyo, Japan). Mitotic cells were identified as those in prophase, metaphase, anaphase, and telophase during mitosis. Mitotic figures indistinguishable from nuclear changes due to apoptosis were excluded from the count (Figure 1).

The expression count of Cyclin A2, Cyclin B1, Cyclin E, or PHH3 was similarly assessed in 10 HPF hot spots. Immunohistochemically positive cells were identified as cells stained intensely. For the quantification of Ki-67 or Survivin, images of hot spots were captured with a microscope digital camera system (Olympus, DP70; Olympus Corporation, Tokyo, Japan), and tumor cells expressing Ki-67 or Survivin were quantified as a percentage of the total number of tumor cells (labeling index) using the e-Count3 software package (e-Path Inc., Fujisawa, Japan). For the determination of the labeling index, 1,000-2,000 tumor cells were examined. For the quantification of mitosis, Cyclin A2, Cyclin B1, Cyclin E, or PHH3, the number of mitotic tumor cells or immunohistochemically positive tumor cells in 10 HPF of hot spots was assessed, as these tumor cells were few and unevenly distributed. In contrast, tumor cells expressing Ki-67 or Survivin were assessed by labeling index (percentage of these tumor cells in total tumor cells) in sections with hot spots, as these cells were relatively numerous.

Statistical analysis

Statistical analyses were performed using the BellCurve Excel software package, version 322 (Social Survey Research Information Co., Ltd., Tokyo, Japan). Cutoff value, sensitivity and specificity, and p-value for the mitotic count or each examined proliferative marker were determined using the receiver operator characteristic (ROC) analysis findings.

## Results

The counts of mitotic cells and the expression of Cyclin A2, Cyclin B1, Cyclin E, or PHH3 (number/10 HPF hot spots), as well as the labeling index (%) of Ki-67 or Survivin for each phyllodes tumor, are presented in Table 3.

**Table 3:** Mitotic count (No./10 HPF), expression count (No./10 HPF) of Cyclin A2, Cyclin B1, Cyclin E, and PHH3, labeling indices (%) of Ki-67 and Survivin, and a maximum diameter of each benign, borderline, or malignant phyllodes tumor. PHH3: phospho-histone H3; HPF: high-power field

S. No.	Mitotic count	Cyclin A2 count	Cyclin B1 count	Cyclin E count	PHH3 count	Ki-67 (%)	Survivin (%)	Diameter (cm)
Benign phyllodes tumors								
1	1	1	2	1	1	27.3	36	0.7
2	1	0	6	0	1	10.2	42	9.5
3	1	0	1	0	1	7.2	31.1	5.1
4	3	1	7	0	3	18.2	47.7	2.3
5	1	1	7	0	3	2.3	28.7	2.2
6	2	2	2	0	2	3.4	36.7	4
7	2	1	0	0	2	13.8	32.5	1.6
8	2	2	3	0	2	8	31.1	2.5
9	1	1	7	0	1	16.2	21.7	2
10	2	0	7	0	2	13.8	40.1	1
11	5	4	5	0	5	11.1	34.6	4.4
12	1	2	0	0	0	4.9	43.7	2
13	1	0	5	0	1	0.3	16.7	2.2
14	1	0	0	1	0	2.7	10.7	2
15	1	0	0	0	0	1.5	29.4	3
16	1	0	3	0	1	8.6	31.6	5.1
17	1	1	3	0	1	1.7	19.3	18
18	3	0	0	0	0	4.9	37.2	2
19	3	0	3	0	1	10.6	44.5	2.6
20	2	1	3	0	1	10.5	46.7	4.3
21	1	0	3	0	0	0.6	0.2	3.5
22	0	0	3	0	0	4.3	29.6	4.2
23	3	0	0	0	0	5.5	41	5.7
24	2	0	0	0	0	1.7	41.7	13
25	1	2	3	0	0	3	29.1	18
26	1	1	0	0	1	1.8	4.4	10
27	2	1	1	0	1	6.7	38.3	2
28	0	7	2	0	0	1.8	10.8	0.8
29	1	0	3	0	0	3.7	35.5	1.4
30	0	0	0	0	0	2.2	36.4	2
31	3	1	1	0	0	2.6	43.4	5
32	0	1	0	0	0	2.1	37.7	6
33	1	1	3	0	3	1.8	12.6	3.6
34	0	0	5	0	1	10.5	16.3	3.7
35	1	1	1	0	0	3.2	26.8	4
36	2	2	1	0	4	6.5	23.3	5
37	5	1	8	0	0	7.1	25	2.4
38	1	0	4	0	0	5.6	17.6	4
39	1	0	5	0	3	4.7	52.2	2
40	1	1	3	0	0	4.2	29.2	2.1
41	1	0	2	0	1	2.4	23.8	2.4
42	2	0	1	0	0	3.5	33.7	4
43	2	0	3	0	2	1.7	13	3
44	2	2	3	0	1	5.4	20.8	2.4
45	0	0	2	0	5	1.7	19.8	2.5
46	4	0	10	0	2	10.4	29	7
47	1	0	6	0	3	8.1	26.6	6.5
Borderline phyllodes tumors								
1	3	1	0	0	1	5.8	41.7	3.8
2	2	0	4	0	2	10	35.6	2.3
3	1	0	1	0	2	12	49	7.5
4	1	1	5	0	1	3.8	34.5	4.2
5	1	1	1	0	3	5.6	34.6	5
6	2	0	4	0	4	2.6	34.4	15
7	2	0	6	2	8	5.6	39.1	6
8	5	1	5	0	8	3.1	39.7	6
9	1	1	2	0	2	9.1	50	1.5
10	3	0	2	0	0	4.5	50	3
11	0	0	3	0	9	3.2	38.7	1.9
12	0	0	12	0	2	2.2	23.1	2.4
13	3	0	1	0	9	4.6	48.4	3.3
Malignant phyllodes tumors								
1	20	7	13	0	20	38.1	59.3	9.5
2	5	7	5	0	5	35.7	14.8	8.4
3	10	1	0	6	5	17	45.5	1.5
4	5	1	26	0	5	8.6	69.5	11.5
5	5	3	2	0	6	10.5	19.3	2.5
6	12	6	11	0	14	13.5	52.3	8
7	6	6	7	0	2	14.8	46.3	4.5
8	21	7	9	0	13	17	43.4	10
9	7	22	20	0	72	20.5	41.5	2.8
10	7	36	13	0	35	16.3	50	6
11	7	1	10	0	28	20.8	50.5	4.8
12	7	1	3	0	20	25	53.3	13.5
13	5	2	10	0	52	12.1	37.9	2.9
14	8	3	11	0	64	6.5	3.3	12
15	9	2	4	0	6	21.5	35	4.5
16	11	2	2	0	5	38.9	99.9	10

Results showing cutoff value, sensitivity and specificity, and positive predictive value (PPV) for mitotic count, Cyclin A2, Cyclin B1, Cyclin E, and PHH3 count in 10 HPF, as well as expression rate (%) for Ki-67 and Survivin, related to the differentiation of phyllodes tumors, are presented in Table 4. The cutoff value for the mitotic count to differentiate malignant phyllodes tumors from benign phyllodes tumors was determined to be 5/10 HPF, resulting in a sensitivity of 1.0 and specificity of 0.9574 (Table 4A). To differentiate malignant phyllodes tumors from both benign and borderline phyllodes tumors, the established cutoff value for the mitotic count was 4/10 HPF, with a sensitivity of 0.95 and specificity of 1.0 (Table 4B). To differentiate malignant phyllodes tumors from borderline phyllodes tumors, the cutoff value for the mitotic count was set at 3/10 HPF, yielding a sensitivity of 0.92 and specificity of 1.0 (Table 4C). Across all three differentiation categories, the mitotic count demonstrated the highest sensitivity and specificity, with both values exceeding 0.9. The PPV for the mitotic count in diagnosing malignant phyllodes tumors was greater than 0.8 in all three differentiation categories (Table 4A-4C).

**Table 4:** Cutoff, sensitivity and specificity, p-value, and positive predictive value (PPV) for mitosis and various proliferative markers. *: PPV of borderline phyllodes tumors; Count: number of cells/10 high-power fields PHH3: phospho-histone H3; PPV: positive predictive value

(A) Benign and malignant phyllodes tumors							
	Mitotic count	Cyclin A2 count	Cyclin B1 count	Cyclin E count	PHH3 count	Ki-67 (%)	Survivin (%)
Cutoff	5	1	8	1	4	12.1	41.5
Sensitivity	1	0.83	0.98	1	0.96	0.81	0.69
Specificity	0.96	0.75	0.56	0.06	0.94	0.89	0.83
p-value	p < 0.05	p < 0.05	p < 0.05	p > 0.05	p < 0.05	p < 0.05	p < 0.05
PPV*	0.89	0.41	0.82	0.33	0.83	0.78	0.58
(B) Both benign/borderline phyllodes tumors and malignant phyllodes tumors							
	Mitotic count	Cyclin A2 count	Cyclin B1 count	Cyclin E count	PHH3 count	Ki-67 (%)	Survivin (%)
Cutoff	4	1	8	2	4	12	41
Sensitivity	0.95	0.87	0.97	1	0.9	0.9153	0.7833
Specificity	1	0.75	0.56	0.06	0.94	0.8125	0.6875
p-value	p < 0.05	p < 0.05	p < 0.05	p > 0.05	p < 0.05	p < 0.05	p < 0.05
PPV*	0.8	0.36	0.75	0.5	0.65	0.684	0.44
(C) Borderline phyllodes tumors and malignant phyllodes tumors							
	Mitotic count	Cyclin A2 count	Cyclin B1 count	Cyclin E count	PHH3 count	Ki-67 (%)	Survivin (%)
Cutoff	3	1.64	6	2	4	12	50
Sensitivity	0.92	1	0.92	0.08	0.69	1	1
Specificity	1	0.75	0.63	0.94	0.62	0.8125	0.8125
p-value	p < 0.05	p < 0.05	p < 0.05	p > 0.05	p < 0.05	p < 0.05	p > 0.05
PPV*	0.8	1	0.83	0.5	0.83	0.929	0.778
(D) Benign and borderline phyllodes tumors							
	Mitotic count	Cyclin A2 count	Cyclin B1 count	Cyclin E count	PHH3 count	Ki-67 (%)	Survivin (%)
Cutoff	2	2	3	1	1	3	33.7
Sensitivity	0.83	0.17	0.72	1	0.7	0.3617	0.617
Specificity	0.31	1	0.46	0.08	0.77	0.9167	0.9231
p-value	p > 0.05	p > 0.05	p > 0.05	p > 0.05	p < 0.05	p > 0.05	p < 0.05
PPV*	0.27	0	0.21	0.33	0.3	0.262	0.387

PHH3 count also showed sensitivity and specificity greater than 0.9 in differentiating malignant phyllodes tumors from benign phyllodes tumors and from both benign and borderline phyllodes tumors (Table 4A-4B). However, PHH3 count showed both sensitivity and specificity lower than 0.7 in differentiating malignant phyllodes tumors from borderline phyllodes tumors. The PPV for PHH3 count in diagnosing malignant phyllodes tumors was 0.83 when differentiating from benign or borderline phyllodes tumors, but 0.65 when differentiating from both benign and borderline phyllodes tumors.

In all three differentiation categories, no other proliferative markers, including Cyclin A2, Cyclin B1, Cyclin E, Ki-67, and Survivin, demonstrated superior sensitivity and specificity at their respective cutoff values compared to the mitotic count (Table 4A-4C).

For differentiation between benign and borderline tumors, the cutoff value for the mitotic count was set at 2/10 HPF, resulting in a sensitivity of 0.83 and specificity of 0.31, indicating low specificity (Table 4D). Similarly, no proliferative marker, except the PHH3 count, demonstrated both high sensitivity and specificity at the specified cutoff value. While the PHH3 count showed relatively high sensitivity and specificity, both were less than 0.8. None of the proliferative indices, including mitotic count and PHH3 count, exhibited a PPV for borderline phyllodes tumors greater than 0.4.

## Discussion

Distinguishing malignant phyllodes tumors from benign and borderline phyllodes tumors is essential due to the disparate clinical behaviors of these entities, as benign and borderline tumors rarely recur or metastasize, while malignant tumors exhibit higher frequencies of recurrence and metastasis [1,2]. The WHO classification outlines histologic criteria for differentiation, including stromal cellularity, stromal atypia, mitotic activity, and stromal overgrowth, with mitotic count specified as an indicator of proliferative capacity [1]. In the present study, the mitotic cycle-related proteins Ki-67, Survivin, Cyclin A2, Cyclin B1, Cyclin E, and PHH3 were examined to determine their usefulness as indices of proliferative potential, in addition to mitotic count. The results revealed mitotic count as the best index based on sensitivity and specificity, and PPV for differentiating a malignant phyllodes tumor from a benign or borderline phyllodes tumor, as well as from both benign and borderline phyllodes tumors.

PHH3 count in phyllodes tumors, particularly in borderline and malignant tumors, has been reported to correlate well with mitotic count [6]. In line with this, the sensitivity and specificity of PHH3 count at the cutoff value determined by ROC analysis for differentiating malignant phyllodes tumors from benign phyllodes tumors, as well as from both benign and borderline tumors, were relatively high (greater than 0.9). However, the sensitivity and specificity of PHH3 count in differentiating malignant phyllodes tumors from borderline phyllodes tumors were much lower compared to mitotic count. Additionally, the PPV of malignant phyllodes tumors was significantly lower for PHH3 count than for mitotic count when differentiating between malignant phyllodes tumors and both benign and borderline phyllodes tumors. Therefore, mitotic count appears to be a more reliable marker than PHH3 count for distinguishing malignant phyllodes tumors from benign or borderline phyllodes tumors, or both.

None of the markers examined, including mitotic count, demonstrated high levels of both sensitivity and specificity for differentiating between benign and borderline phyllodes tumors. Furthermore, the PPV for borderline phyllodes tumors was below 0.4 for all proliferative markers. Those findings suggest that assessing proliferative potential may not be critical for distinguishing borderline phyllodes tumors from benign phyllodes tumors, thus emphasizing the importance of factors other than proliferative potential in the differentiation process. Meanwhile, the WHO classification criteria define mitotic count for benign, borderline, and malignant phyllodes tumors as less than 5/10 HPF, 5-10/10 HPF, and above 10/10 HPF, respectively, with a cutoff value of 5/10 HPF for distinguishing between benign and borderline status. The present findings suggest that the significance of this cutoff value warrants reevaluation in terms of sensitivity and specificity.

Observer variability for mitotic count has been acknowledged and attributed to differences in image perception and high magnification area, as well as measurement site variation. Correcting for differences in the high magnification area between microscopes can be achieved by presenting mitotic count per area. Although the WHO classification uses the number/10 HPF as the cutoff value, the area of 1 HPF varies among microscopes; thus, it is advisable to state the area by 1 HPF for conversion to per mm². In the present study, 10 HPF corresponded to 2.38 mm². To address differences in the perception of mitotic images among observers, it is crucial to utilize mitotic images of nuclei in the prophase, metaphase, anaphase, and telophase in mitosis, which are recognizable in specimens stained with hematoxylin and eosin, and to avoid confusion between mitotic cells and apoptotic cells showing nuclear condensation, cytoplasmic eosinophilia, and gaps with the surrounding area due to cell shrinkage [14,15]. As noted above, standardization of mitotic count evaluation methods is crucial when determining cutoff values.

In the WHO classification, the cutoff value noted for mitotic count (number/10 HPF) for differentiating malignant phyllodes tumors from borderline phyllodes tumors is 10. However, that cutoff value was determined in the present study to be 4/10 HPF. This disparity may be attributed to the strict definition of the number of mitotic cells used for this analysis, as well as the lack of a definitive size of one field of view under a microscope in the WHO criteria for mitotic cell count. Therefore, further studies with a substantial tumor number and a strict definition of mitosis, expressing mitotic count as a number per area (mm²), are required to determine the cutoff value.

## Conclusions

The present findings indicate mitotic count as the most reliable indicator of proliferative potential and show its ability to serve as a key factor for distinguishing malignant from benign and/or borderline phyllodes tumors of the breast. Nonetheless, it is evident that criteria other than the proliferation index can play a crucial role in the differentiation between benign and borderline phyllodes tumors.
